# White matter network underlying semantic processing: evidence from stroke patients

**DOI:** 10.1093/braincomms/fcae058

**Published:** 2024-02-21

**Authors:** Xiangyue Xiao, Zhicai Dong, Mingyan Yu, Junhua Ding, Maolin Zhang, Sara Cruz, Zaizhu Han, Yan Chen

**Affiliations:** School of Basic Medical Sciences, Hangzhou Normal University, Hangzhou 311121, China; Key Laboratory of Aging and Cancer Biology of Zhejiang Province, School of Basic Medical Sciences, Hangzhou Normal University, Hangzhou 311121, China; School of Basic Medical Sciences, Hangzhou Normal University, Hangzhou 311121, China; Key Laboratory of Aging and Cancer Biology of Zhejiang Province, School of Basic Medical Sciences, Hangzhou Normal University, Hangzhou 311121, China; School of Basic Medical Sciences, Hangzhou Normal University, Hangzhou 311121, China; Key Laboratory of Aging and Cancer Biology of Zhejiang Province, School of Basic Medical Sciences, Hangzhou Normal University, Hangzhou 311121, China; State Key Laboratory of Cognitive Neuroscience and Learning & IDG/McGovern Institute for Brain Research, Beijing Normal University, Beijing 100875, China; Department of Psychology, University of Edinburgh, Edinburgh EH8 9YL, UK; School of Basic Medical Sciences, Hangzhou Normal University, Hangzhou 311121, China; The Psychology for Development Research Center, Lusiada University Porto, Porto 4100-348, Portugal; State Key Laboratory of Cognitive Neuroscience and Learning & IDG/McGovern Institute for Brain Research, Beijing Normal University, Beijing 100875, China; School of Basic Medical Sciences, Hangzhou Normal University, Hangzhou 311121, China; Key Laboratory of Aging and Cancer Biology of Zhejiang Province, School of Basic Medical Sciences, Hangzhou Normal University, Hangzhou 311121, China; State Key Laboratory of Cognitive Neuroscience and Learning & IDG/McGovern Institute for Brain Research, Beijing Normal University, Beijing 100875, China

**Keywords:** white matter network, semantic processing, semantic hub, modality-specific connection, stroke

## Abstract

The hub-and-spoke theory of semantic representation fractionates the neural underpinning of semantic knowledge into two essential components: the sensorimotor modality-specific regions and a crucially important semantic hub region. Our previous study in patients with semantic dementia has found that the hub region is located in the left fusiform gyrus. However, because this region is located within the brain damage in patients with semantic dementia, it is not clear whether the semantic deficit is caused by structural damage to the hub region itself or by its disconnection from other brain regions. Stroke patients do not have any damage to the left fusiform gyrus, but exhibit amodal and modality-specific deficits in semantic processing. Therefore, in this study, we validated the semantic hub region from a brain network perspective in 79 stroke patients and explored the white matter connections associated with it. First, we collected data of diffusion-weighted imaging and behavioural performance on general semantic tasks and modality-specific semantic tasks (assessing object knowledge on form, colour, motion, sound, manipulation and function). We then used correlation and regression analyses to examine the association between the nodal degree values of brain regions in the whole-brain structural network and general semantic performance in the stroke patients. The results revealed that the connectivity of the left fusiform gyrus significantly predicted general semantic performance, indicating that this region is the semantic hub. To identify the semantic-relevant connections of the semantic hub, we then correlated the white matter integrity values of each tract connected to the left fusiform gyrus separately with performance on general and modality-specific semantic processing. We found that the hub region accomplished general semantic processing through white matter connections with the left superior temporal pole, middle temporal gyrus, inferior temporal gyrus and hippocampus. The connectivity between the hub region and the left hippocampus, superior temporal pole, middle temporal gyrus, inferior temporal gyrus and parahippocampal gyrus was differentially involved in object form, colour, motion, sound, manipulation and function processing. After statistically removing the effects of potential confounding variables (i.e. whole-brain lesion volume, lesion volume of regions of interest and performance on non-semantic control tasks), the observed effects remained significant. Together, our findings support the role of the left fusiform gyrus as a semantic hub region in stroke patients and reveal its crucial connectivity in the network. This study provides new insights and evidence for the neuroanatomical organization of semantic memory in the human brain.

## Teaser

Which white matter network underlies semantic knowledge processing? Xiao *et al*. identified the left fusiform gyrus as the semantic hub in stroke patients. This hub works with four regions for general semantic processing and functions with other regions for modality-specific semantic processing.

## Graphical Abstract

**Graphical Abstract:**
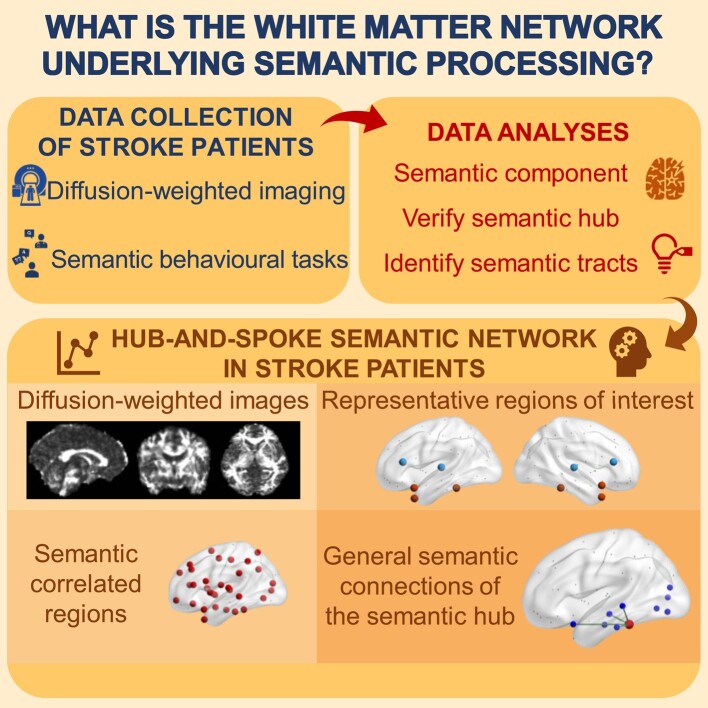

## Introduction

The ability to understand sounds and the meaning of words, to communicate with objects and other people and to interact with the environment all depend on our ability to use semantic knowledge. According to Tulving,^1^ semantic memory is the component of human memory that corresponds to general knowledge about objects, word meanings, facts and people, without connection to a specific time or place. Semantic cognition refers to the group of neurocognitive processes that underpin behaviours with semantic undertones.^2-6^ If the semantic system of the brain is damaged for various reasons, we will not be able to perceive the world and communicate with people properly.

Investigating the representation of semantic knowledge in the brain offers clarification of semantic processing. The hub-and-spoke theory,^7^ which has received considerable support in the literature, divides the neural underpinning of semantic knowledge into two essential components: (i) multiple modality-specific regions that serve as spokes to represent modality-specific information about concepts and (ii) a crucially important semantic hub region that gathers information from distributed spoke regions to form an amodal semantic representation.^6-9^ Previous studies have suggested that the anterior temporal lobe (ATL) is the hub that aggregates neural processing from various sources.^8-14^ However, which area of the ATL is the hub region remains controversial because it includes several areas [such as the temporal pole, fusiform gyrus (FFG), superior temporal gyrus, middle temporal gyrus and inferior temporal gyrus]. Previous studies have also shown that patients with semantic deficits may exhibit impairments in certain modalities of knowledge such as form,^15,16^ colour,^15-19^ sound^20,21^ and function.^22,23^ Such modality-specific semantic impairments may result from varying degrees of damage in distributed brain regions outside the ATL.^8^ These studies have provided favourable support for the hub-and-spoke representation theory.

In a previous study, we investigated the correlation between the nodal degree of each brain region in the structural network and semantic performance in patients with semantic dementia.^18^ The results revealed that the left FFG was a hub region for semantics and that the connections between this hub region and nine other brain regions were related to the general and modality-specific semantic deficits of the subjects. However, the hub region found in our study is located in the area of the brain with the most severe damage in patients with semantic dementia.^18^ Therefore, it is difficult to rule out the possibility that its unique role was only due to its large variation. The need to be wary of such false positives due to overestimation of apparently damaged brain areas was also pointed out by Visser *et al.*^24^ Furthermore, because local damage and disconnections of the hub region are concurrent and inseparable in patients with semantic dementia, it is impossible to determine whether the semantic deficits are the result of structural damage in the local hub region or of disconnections between the hub and other brain regions.

Thus, for further exploration, it is necessary to select another type of brain-injured patients who have no significant damage in the hub region but still exhibit semantic impairment. Stroke is a common brain medical condition that can also lead to multimodal semantic deficits.^25^ Using resting-state functional MRI, Zhao *et al.*^10^ identified the ATL as a central hub for semantic processing in stroke patients, and that reduced nodal degree values in this network served as predictive indicators of deficits in general and modality-specific semantic performance. PET studies in aphasic stroke patients have shown that reduced activation in specific ATL regions is associated with semantic deficits and that ATL connectivity correlates with functional outcome.^12,14^ Some case reports have suggested that stroke patients also experience difficulties in modality-specific semantic processing. For example, damage to distributed spoke regions can lead to impairments in specific object colour knowledge,^15,17,19^ as well as functional knowledge deficits related to living things.^22^ Unlike semantic dementia, stroke is not a neurodegenerative disease, but rather a rupture of a blood vessel in the brain or a cerebrovascular embolism that causes localized neurological deficits and thus symptoms of aphasia.^26^ It is commonly caused by damage to several main cerebral arteries,^27,28^ which usually do not include the ventral part of the temporal lobe. Accordingly, the pattern of brain injury in stroke differs significantly from that in semantic dementia. Therefore, stroke serves as an ideal lesion model to complement the neural organization model of semantic memory generated in semantic dementia. A detailed study of the structural network and semantic performance in stroke patients can answer the following key questions. First, is the hub region of the semantic network located in the left FFG as identified in our previous research?^18^ Second, is semantic processing determined solely by the structural integrity of the hub region or its connection in the network? Previous research has identified semantic-relevant connections in patients with semantic dementia,^18^ but could these connections serve similar functions in patients with different patterns of brain lesion? Are there new connections through which the hub region might work with other brain regions for general or modality-specific semantic functions?

Therefore, the aim of the present study was 2-fold: (i) to confirm the hub region described in the semantic dementia study^18^ and (ii) to identify the semantic white matter tracts connected to the semantic hub based on 79 stroke patients. To verify the semantic hub in stroke patients, we first correlated general semantic performance with the nodal degree value of each region in the whole-brain white matter network in stroke patients. We then performed regression analyses on 10 specific brain regions, including the left FFG and other putatively semantic relevant and irrelevant regions, to locate the semantic hub. Finally, to explore general and modality-specific semantic white matter connectivity, we correlated general and modality-specific semantic performance with the integrity metrics of each white matter tract connected to the semantic hub.

## Materials and methods

### Participants

Patients with stroke and healthy control subjects took part in the current study. All participants were right handed, native Chinese speakers and provided written informed consent. This study was approved by the Research Ethics Committee of the School of Basic Medical Sciences, Hangzhou Normal University.

### Stroke patients

Seventy-nine patients with stroke (65 males) were recruited from the China Rehabilitation Research Centre. They met the following inclusion criteria: no previous brain injury; no other neurological or psychiatric disease; at least 1-month post-onset [mean = 4.78 months; standard deviation (SD) = 3.92; range: 1–24 months]; no history of alcoholism; normal or corrected-to-normal hearing and vision; and able to follow task instructions. The background information of the stroke patients is shown in Supplementary Table 1. The patients’ mean age was 46.90 years (SD = 11.24; range: 20–74 years), and the mean years of formal education was 13.47 (SD = 3.96; range: 6–19). Forty-nine patients suffered from an ischaemic stroke, and 30 patients suffered from a haemorrhagic stroke. Most of them had a left hemisphere stroke (*n* = 48), and the others had a right hemisphere stroke (*n* = 31). Neuropsychological testing for Chinese aphasia^29^ showed that 71 patients presented symptoms of aphasia (motor aphasia, *n* = 15; sensory aphasia, *n* = 10; conduction aphasia, *n* = 5; anomic aphasia, *n* = 10; global aphasia, *n* = 26; subcortical aphasia, *n* = 4; dysgraphia, *n* = 1). The patients’ mean score on the Chinese version of the Mini-Mental State Examination (MMSE)^30^ was 20.99 ± 8.28.

### Healthy controls

Forty-one healthy control subjects (22 males) participated in the study. They were recruited from patients’ acquaintances and the local community. The subjects’ mean age was 50.54 years (SD = 10.82; range: 26–72 years), and their mean years of formal education was 12.54 (SD = 2.89; range: 6–22 years). They also had normal or corrected-to-normal hearing and vision and no history of alcoholism, brain injury, psychiatric or neurological disease. Their MMSE mean score was 28.32 ± 1.35.

The stroke patients and healthy controls were comparable in age and educational years (*P* > 0.05), while the patients had lower MMSE scores than healthy controls (*t* = −8.12, *P* < 0.001).

### Behavioural data collection

Behavioural data were collected for both stroke patients and healthy controls. Patients with language impairments were given additional instructions to ensure that they understood the tasks (see details in the Supplementary material). The methods used to collect the behavioural data have been previously described.^4,10,18^ First, the subjects’ general semantic processing ability was assessed using six tasks: oral picture naming (140 items, subjects were instructed to name the objects that appeared on a screen); oral sound naming (36 items, subjects listened to the sounds from earphones and had to name the objects that produced the sounds); picture-associative matching (70 items, subjects were instructed to identify which of the two object pictures at the bottom of the screen was semantically closer to the picture at the top of the screen); word-associative matching (70 items, this task is identical to the picture-associative matching task except that the object pictures were replaced by their written names); word–picture verification (70 items, subjects were shown a picture and a word and had to determine whether the word and the picture matched); and naming to definition (70 items, subjects were instructed to name the objects whose definitions were presented visually and aurally). All these tasks tested the general aspects of semantic knowledge but varied in the modalities of input and output. Afterwards, 12 modality-specific semantic tasks were administered to assess the subjects’ processing ability on six specific sensorimotor modalities of objects (form, colour, motion, sound, manipulation and function). Details can be found in the Supplementary material. Finally, three non-semantic control tasks (visual perception, sound perception and number proximity matching) were conducted to control for the influence of non-semantic processing abilities Table 1; Supplementary material).

**Table 1:** Behavioural performance of the participants

Tasks	Raw accuracy: mean (standard deviation)		Corrected *t*-score (standard deviation) of patients
	Healthy controls	Stroke patients	
General semantic task			
Oral picture naming	94% (4%)	67% (29%)^a^	−6.51 (6.93)
Oral sound naming	82% (12%)	54% (29%)^a^	−2.85 (2.66)
Picture-associative matching	94% (4%)	87% (9%)^a^	−1.45 (2.00)
Word-associative matching	96% (3%)	88% (14%)^a^	−2.76 (4.60)
Word–picture verification	97% (3%)	89% (12%)^a^	−2.94 (4.42)
Naming to definition	89% (7%)	61% (33%)^a^	−4.91 (5.43)
Modality-specific semantic task			
Verbal task			
Form matching	93% (6%)	85% (14%)^a^	−1.67 (2.50)
Colour matching	94% (5%)	83% (14%)^a^	−2.65 (3.21)
Motion matching	93% (5%)	84% (11%)^a^	−2.06 (2.30)
Sound matching	86% (9%)	77% (13%)^a^	−1.28 (1.50)
Manipulation matching	93% (5%)	79% (16%)^a^	−3.38 (3.49)
Function matching	98% (3%)	89% (15%)^a^	−3.04 (5.41)
Non-verbal task			
Form verification	83% (9%)	74% (13%)^a^	−1.39 (1.46)
Colour verification	77% (12%)	62% (16%)^a^	−1.37 (1.26)
Motion verification	69% (14%)	51% (18%)^a^	−1.63 (1.19)
Sound verification	85% (8%)	71% (15%)^a^	−2.11 (1.84)
Manipulation matching	89% (8%)	79% (14%)^a^	−1.41 (1.69)
Function matching	96% (4%)	89% (11%)^a^	−1.53 (2.38)
Non-semantic control task			
Visual perception	86% (11%)	83% (11%)^a^	−1.21 (1.84)
Sound perception	84% (16%)	79% (18%)^a^	−1.77 (2.14)
Number proximity matching	86% (21%)	83% (23%)^a^	−0.49 (1.36)

Group difference of raw accuracies was compared between the patient and control groups using two-sample *t*-test.

^a^FDR-corrected *q* < 0.01.

### Behavioural data preprocessing

As the patients varied considerably in their demographic properties, their raw scores on the behavioural tasks may not meaningfully reflect the degree of deficit. Therefore, we used the single case-to-control method proposed by Crawford and Garthwaite^31^ to correct the patients’ behavioural scores by considering the demographic information and performance of the healthy controls. Specifically, a regression model was performed for each task based on the characteristics of the healthy group, with age, gender and education level as independent variables and the subjects’ accuracy scores as dependent variables. The demographic variables of each patient were then entered in the model, and a predicted accuracy score was obtained. Finally, by dividing the discrepancy between the observed and predicted accuracy by the corrected standard error of the estimate, the patients’ corrected *t*-scores were determined.

To measure the patients’ general semantic cognitive ability, their *t*-scores on the six general semantic tasks and three control tasks were put into SPSS 20.0 for principal component analysis (PCA).^32^ The component with relatively high loading weights on tasks in which semantic processing is highly relevant (i.e. the six general semantic tasks) relative to those tasks in which semantic processing is not central (i.e. the three control tasks) was considered as the semantic component. The scores corresponding to this component were considered to reflect the general semantic processing of the stroke patients.

To assess the patients’ modality-specific semantic ability, we calculated the mean of the corrected *t*-scores of the verbal and non-verbal tasks in each semantic modality. The six modality-specific semantic scores obtained were used in the following analyses.

### Imaging data collection

Each subject was scanned with a 1.5T GE SIGNA EXCITE scanner at the China Rehabilitation Research Centre. We collected three types of images: 3D T_1_-weighted images, T_2_-weighted fluid-attenuated inversion recovery (FLAIR) images and diffusion-weighted images (DWIs). Details on the sequences of 3D T_1_-weighted and T_2_-weighted FLAIR images can be found in the Supplementary material. Diffusion-weighted imaging comprised two separate sequences with different diffusion weighting direction sets. The parameters for the first acquisition were as follows: repetition time = 13 000 ms, echo time = 69.3 ms, flip angle = 90°, matrix size = 128 × 128, field of view = 250 mm × 250 mm, slice number = 53 slices, slice thickness = 2.6 mm, voxel size = 1.95 mm × 1.95 mm × 2.6 mm and direction number = 15 directions. The other acquisition had the same parameters but included 17 different directions. In each DWI sequence, the first two images were *b* = 0 image, and the remaining images were acquired with a *b*-value of 1000 s/mm^2^. All the sequences except the T_2_ FLAIR images were scanned twice to improve the quality of the images.

### Imaging data preprocessing

**Figure 1:**
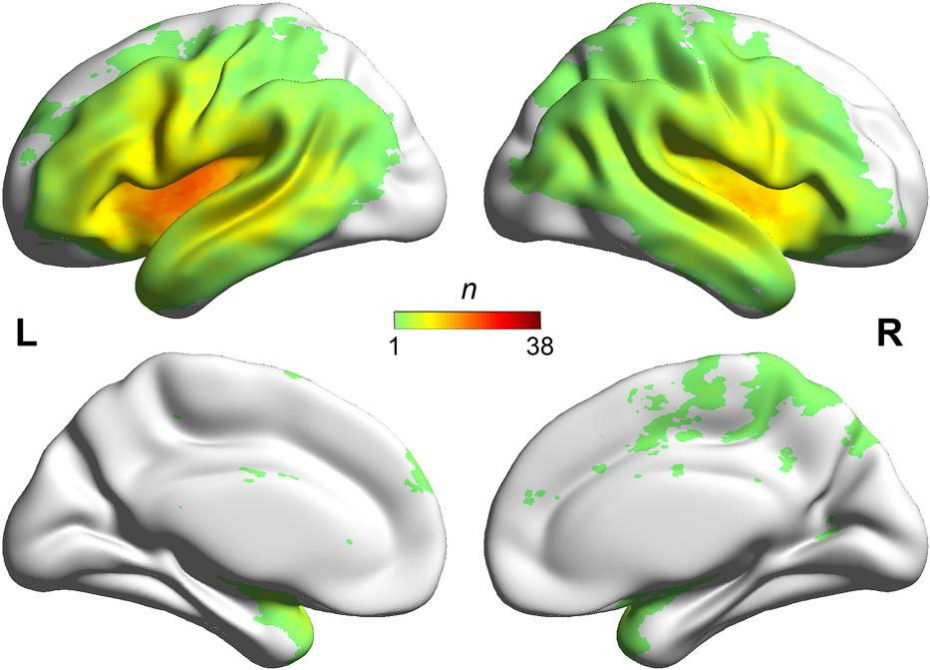
Lesion overlap map of the 79 stroke patients (the *n*-value of each voxel denotes the number of patients with lesion).

We first coregistered the two T_1_ images in the native space using a trilinear interpolation method in SPM5 and then averaged them. The T_2_ FLAIR images were then coregistered and resliced to the averaged T_1_ images. Two trained researchers manually drew each patient’s lesion contour on the averaged T_1_ image, visually referring to the T_2_ FLAIR image. Both researchers independently achieved a reasonable level of interrater reliability with an experienced radiologist during the training phase (mean percentage volume differences of 9% ± 8% and 4% ± 3%, mean percentage of discrepant voxels^33,34^ of 7% ± 4% and 6% ± 2%). Subsequently, the lesion drawing of each patient was double checked by the radiologist. Each patient’s structural images were resliced into a voxel size of 1 mm × 1 mm × 1 mm and then normalized into Talairach space^35^ via the ‘3D Volume Tools’ in BrainVoyager. The affine transformation matrix between the native and Talairach spaces was estimated and further employed to transform the lesion masks into the Talairach space. Finally, the lesion masks were transformed into Montreal Neurological Institute (MNI) space. To illustrate the lesion area of the stroke patients, we superimposed the lesion masks of all the patients in the MNI space and obtained the lesion overlap map (Fig. 1).

The diffusion-weighted imaging data with 15 directions and 17 directions of each stroke patient were first merged into a 4D image and then preprocessed using a pipeline tool for analysing brain diffusion images (PANDA).^36^ First, the skull was removed from the *b* = 0 image with the ‘bet’ command. Next, eddy current distortion and simple head motion were corrected by registering the DWIs to the *b* = 0 image with an affine transformation using the ‘eddy_correct’ command. Afterwards, the diffusion tensor models were built, and individual fractional anisotropy, mean diffusivity, axial diffusivity and radial diffusivity maps were obtained. This step was achieved with the ‘dtifit’ command. Finally, the individual diffusion tensor images in the native space were non-linearly registered to the MNI space with target voxel size of 2 mm × 2 mm × 2 mm. To do this, the ‘fnirt’ and ‘applywarp’ commands were applied.

### Brain network construction in healthy subjects

For network construction, we adopted the identical approach as described by Chen *et al.*^18^ We used tracts derived from healthy participants as masks on images of stroke patients and extracted integrity values of the tracts in the patients.

The white matter masks of the healthy participants were identical to those used in our previous study.^18^ Specifically, grey matter regions in the automated anatomical labelling (AAL) atlas were defined as network nodes, and we performed deterministic fibre tractography between every two nodes in each healthy subject.^37^ The resulting structural network maps from all the healthy subjects were then overlaid in the MNI space, and a count map was generated. To determine the anatomical connectivity between each node pair, a group-level threshold of voxel value >25% of subjects and cluster size >300 voxels was adopted to the count map.^34^ Finally, 457 tracts passed the threshold.

We used these 457 tracts derived from healthy participants as masks and extracted the mean fractional anisotropy and mean, axial and radial diffusivity values of voxels within each tract for each stroke patient.

### Verifying the semantic hub

Before verifying the semantic hub, we determined the semantic correlated regions. First, we computed the nodal degree value of each AAL region for each diffusion metric of each patient. The degree value was calculated by summing the diffusion metric values of all white matter tracts connected to the node in the whole-brain network. A two-tailed Pearson correlation was then computed to correlate general semantic performance (i.e. the semantic PCA scores) with the nodal degree value of each AAL region in 79 stroke patients. A threshold of *q* < 0.01 with false discovery rate (FDR) was adopted to correct for multiple comparisons.

**Figure 2:**
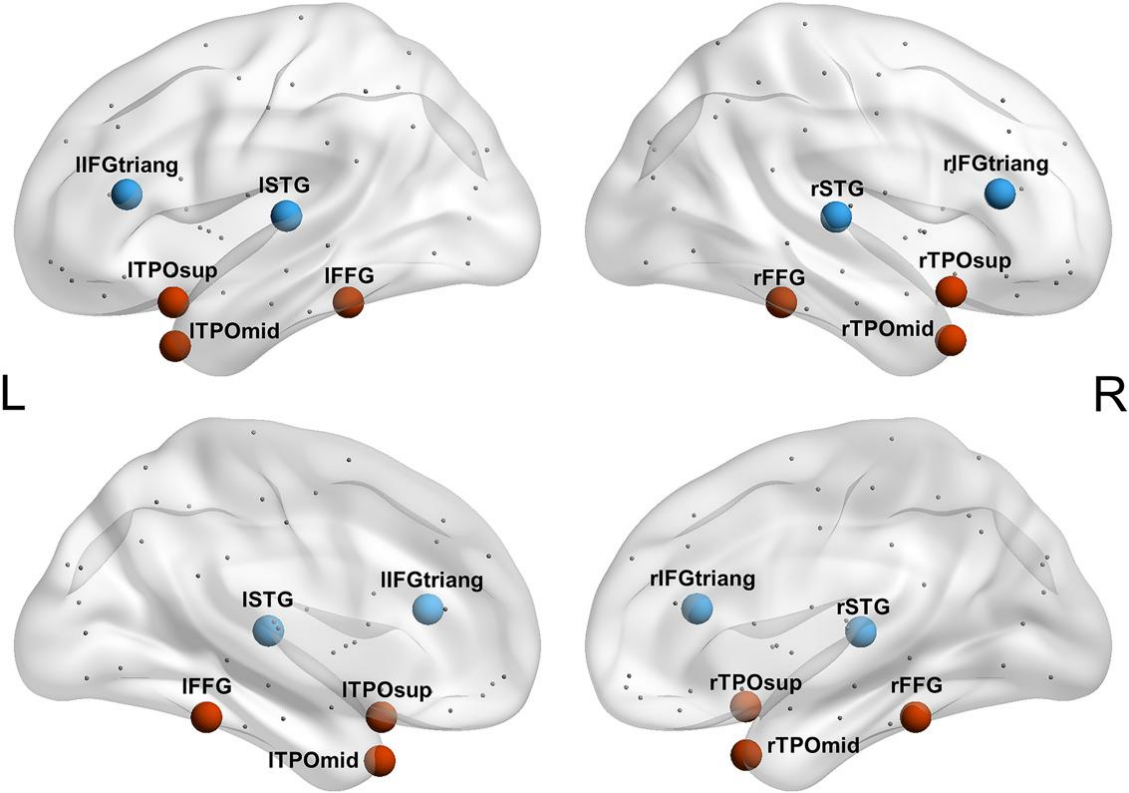
**Ten representative ROIs.** We selected 10 representative ROIs for stepwise regression analyses. They included six putatively semantic relevant AAL regions: bilateral fusiform gyri (lFFG, rFFG), superior temporal poles (lTPOsup, rTPOsup) and middle temporal poles (lTPOmid, rTPOmid), and four putatively irrelevant ones: bilateral superior temporal gyri (lSTG, rSTG) and the triangular part of inferior frontal gyri (lIFGtriang, rIFGtriang).

However, it is possible that the nodal degree of the regions correlated with the semantic scores, yet they were not relevant to semantic functions. This is due to the covariation of the degree values in these regions with the semantic hub, resulting from the degeneration of many tracts that run through the stroke-affected regions. Therefore, to identify the semantic hub, we compared the effects of potential hub regions. Ten representative regions of interest (ROIs) were selected from the correlation results, including six putatively semantic-relevant AAL regions (i.e. bilateral fusiform gyri, superior temporal poles and middle temporal poles) and four putatively irrelevant ones (i.e. bilateral superior temporal gyri and the triangular part of inferior frontal gyri; Fig. 2).^13,18^ The nodal degree values of these regions were then used in a regression analysis to verify the semantic hub. To correct for the potential influence of confounders (i.e. whole-brain lesion volume, calculated as the total number of lesioned voxels across the whole brain; lesion volume of the brain region itself; and performance on the non-semantic control tasks, the corrected *t*-scores of the visual perception, sound perception and number proximity matching tasks), we first calculated the residual for the degree value of each region by regressing out all of the covariates. The resulting values were then entered into stepwise regressions with the semantic PCA scores as the dependent variable. Predictors were accepted into the model with a significance threshold of 0.05. We further performed similar analyses on patients with semantic deficits, whose semantic performance was lower than that of healthy controls, to validate our results.

### Identifying the semantic-relevant tracts of the semantic hub

To investigate how the semantic hub region interacts with other regions for semantic processing, we explored general and modality-specific semantic tracts connected to the semantic hub.

### Identifying the general semantic tracts of the semantic hub

To identify the general semantic tracts of the semantic hub, we examined the relationship between the integrity of white matter tracts connected to the hub and general semantic performance in 79 stroke patients. Specifically, we employed a two-tailed Pearson correlation to examine the correlation between the integrity of each tract of the hub (across four diffusion metrics) and the patients’ semantic PCA scores. The threshold was FDR-corrected *q* < 0.01 and significant for at least two metrics.

Partial correlation analyses were conducted to determine whether the effects of the general semantic tracts were driven by these potential confounders: (i) whole-brain lesion volume; (ii) lesion volumes of the semantic hub and the other node; and (iii) *t*-scores of three non-semantic control tasks. The significance threshold was set at *P* < 0.05.

### Identifying the modality-specific semantic tracts of the semantic hub

To identify the modality-specific semantic tracts of the semantic hub, we examined the Pearson correlation between the integrity of each tract connected to the hub (across four diffusion metrics) and six modality-specific semantic scores in the stroke patients. The threshold was FDR-corrected *q* < 0.01 and significant for at least two metrics.

To confirm the effects of the observed connections, we performed partial correlation analyses with a threshold of *P* < 0.05, excluding the influence of the aforementioned confounders.

### Statistical analysis

For each behavioural task, we performed a two-sample *t*-test on the raw accuracies between the patient and control groups using the SPSS 20.0. The threshold was set at FDR-corrected *q* < 0.01. For the imaging data, stepwise regression analyses in the ‘Verifying the semantic hub’ section were performed using the SPSS 20.0. Two-tailed Pearson correlation analyses in the ‘Verifying the semantic hub’ and ‘Identifying the semantic-relevant tracts of the semantic hub’ sections were performed using the MATLAB R2020b.

## Results

### Behavioural performance of participants

Table 1 shows the participants’ raw accuracies and corrected *t*-scores on the behavioural tasks. The stroke patients exhibited significantly lower raw accuracies in each task compared to the control group (*t* < −2.43; FDR-corrected *q*s < 0.01). The corrected *t*-scores of the patients (<−0.49) also indicated that they had marked impairments in these tasks.

The semantic processing abilities of the stroke patients were determined using PCA based on nine cognitive tasks varying in the degree of semantic involvement and input/output modalities. Three components showing eigenvalues >1 were extracted (Table 2). Component 1 (eigenvalue = 4.22) accounted for 40% of the model variance, with the six general semantic tasks having higher loading weights (0.44–0.93) while the three non-semantic control tasks having lower loading values (−0.19 to 0.30). We therefore labelled this component as the semantic component and derived scores for each patient’s general semantic processing ability based on this component. Component 2 (eigenvalue = 1.51) and Component 3 (eigenvalue = 1.04) were treated as the perceptual and arithmetic components because of their respective heavier loading weight on the visual and sound perception tasks (0.32–0.82) and the number proximity matching task (0.74).

**Table 2:** Loading weight of each task on each component in PCA

Tasks	Semantic component	Perceptual component	Arithmetic component
Oral picture naming	0.92	0.04	0.25
Oral sound naming	0.91	0.03	0.22
Picture-associative matching	0.44	0.59	0.28
Word-associative matching	0.48	0.75	−0.12
Word–picture verification	0.72	0.42	−0.14
Naming to definition	0.93	0.09	0.16
Visual perception	0.01	0.32	0.67
Sound perception	−0.19	0.82	0.23
Number proximity matching	0.30	−0.06	0.74

### Brain damage of stroke patients

Figure 1 illustrates the lesion overlap map of the 79 stroke patients. As shown, the patients exhibited brain lesions predominantly in the bilateral insula and the surrounding areas. Note that the patients did not show damage in the left FFG (Supplementary material and Supplementary Table 2). This allowed us to rule out damage to the left FFG itself in stroke patients and to investigate whether its connectivity (nodal degree values) was still predictive of patient’s semantic performance.

### Verifying the semantic hub region

As depicted in Supplementary Fig. 1, the regions whose nodal degree values correlated with the patients’ semantic performance were widely distributed across the brain (FDR-corrected *q*s < 0.01), as expected. Therefore, we performed the regression analysis to verify whether the semantic hub region was identical to that observed in semantic dementia.^18^ The results showed that the significant factors accounting for the semantic performance of the stroke patients were the nodal degree values of the bilateral FFG (*P* < 0.02, except for the fractional anisotropy model; Table 3).

**Table 3:** Linear regression modelling under four different diffusion metrics in 79 stroke patients

Metric	Predictor	Beta (standardized)	Beta (unstandardized)	*t*	*P*	Adjusted *R*^2^	*F*	Significance
Fractional anisotropy	Right fusiform gyrus	−0.56	−0.58	−5.66	2.5 × 10^−7^	0.33	20.36	8.3 × 10^−8^
	Left triangular part of inferior frontal gyrus	0.46	0.48	4.71	1.1 × 10^−5^			
Mean diffusivity	Right fusiform gyrus	0.44	0.45	4.34	4.3 × 10^−5^	0.22	12.03	2.9 × 10^−5^
	Left fusiform gyrus	−0.26	−0.27	−2.58	1.2 × 10^−2^			
Axial diffusivity	Right fusiform gyrus	0.40	0.41	3.94	1.8 × 10^−4^	0.20	10.50	9.4 × 10^−5^
	Left fusiform gyrus	−0.27	−0.28	−2.63	1.0 × 10^−2^			
Radial diffusivity	Right fusiform gyrus	0.45	0.46	4.46	2.8 × 10^−5^	0.22	12.20	2.5 × 10^−5^
	Left fusiform gyrus	−0.26	−0.27	−2.60	1.1 × 10^−2^			

This table shows the results of the linear regression. Four models have been created based on the nodal degree values of 10 ROIs under different diffusion metrics. Stepwise regression was employed in each of these models to identify variables that can predict semantic performance. Details about the coefficients of the predictors (standardized and unstandardized beta-, *t*-, and *P*-values) and the model (adjusted *R*^2^, *F*- and *P*-values) are shown.

Note that, as shown in Table 3, when the nodal degree values under the fractional anisotropy metric were used as independent variables in the stepwise regression analysis, the beta value for the right FFG was negative. For the other three metrics (mean, axial and radial diffusivity), we consistently found positive beta values for the right FFG, but negative beta values for the left FFG. It is known that in stroke, the fractional anisotropy value of the affected region typically decreases and the diffusivity values increase.^38,39^ This suggests that the nodal degree of the semantic hub region should correlate positively with semantic scores on the fractional anisotropy metric and negatively with scores on the three diffusivity metrics. Therefore, the results suggest that only the left FFG is the semantic hub region in stroke patients. Similarly, the results of validation analyses performed on the subgroup of patients with semantic deficits were highly consistent with these findings, further supporting the role of the left FFG as the semantic hub (Supplementary material).

### Semantic-relevant connectivity of the semantic hub

**Figure 3:**
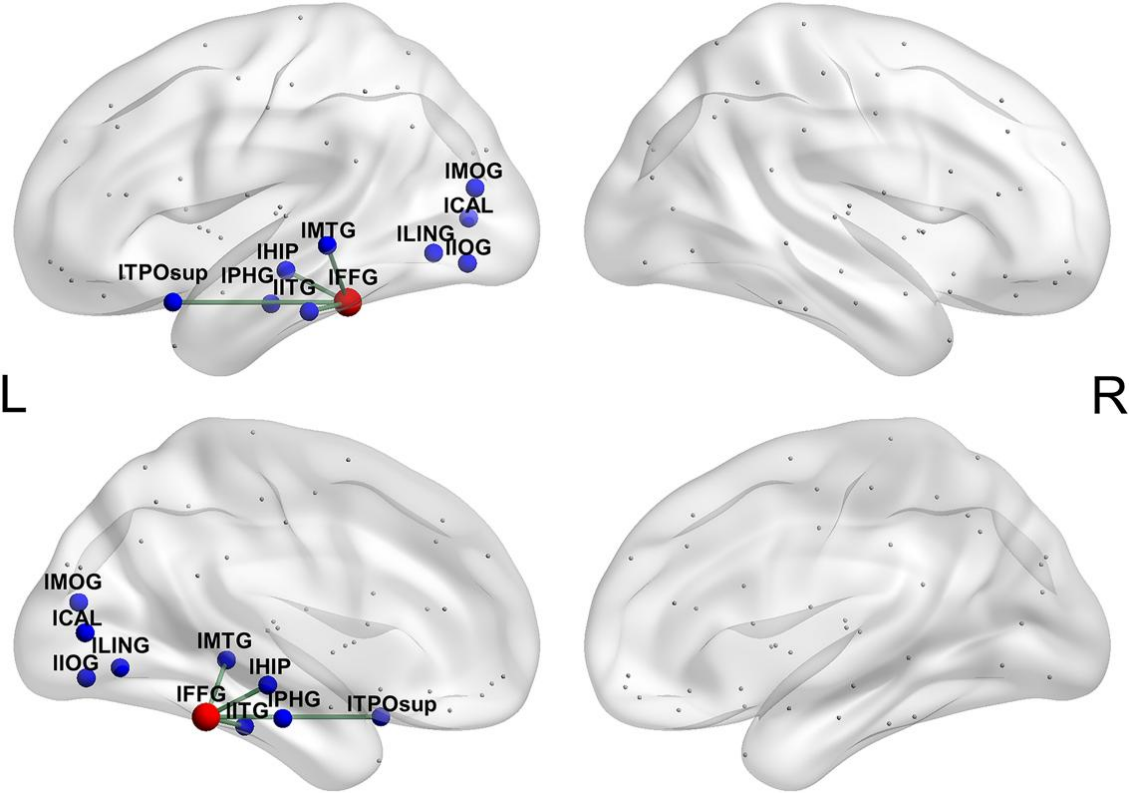
**The general semantic connections of the semantic hub.** The left fusiform in the network was connected to nine regions, namely the left superior temporal pole, hippocampus, parahippocampal gyrus, inferior temporal gyrus, middle temporal gyrus, lingual gyrus, calcarine, inferior occipital gyrus and middle occipital gyrus. Using a two-tailed Pearson correlation, the correlation between the integrity of each tract of the hub and the patients’ semantic PCA scores was analysed. The threshold was FDR-corrected *q* < 0.01 and significant for at least two metrics. The white matter connections between the left fusiform and four brain regions, namely the left superior temporal pole (*r* < −0.32, *P* < 0.005), left middle temporal gyrus (*r* < −0.32, *P* < 0.004), left inferior temporal gyrus (*r* < −0.30, *P* < 0.005) and left hippocampus (*r* < −0.40, *P* < 0.0003), were found to be relevant to the general semantic performance in the 79 stroke patients. lCAL, left calcarine; lFFG, left fusiform gyrus; lHIP, left hippocampus; lIOG, left inferior occipital gyrus; lITG, left inferior temporal gyrus; lLING, left lingual gyrus; lMOG, left middle occipital gyrus; lMTG, left middle temporal gyrus; lPHG, left parahippocampal gyrus; lTPOsup, left superior temporal pole.

The left FFG in the network was connected to nine regions, including the left superior temporal pole, hippocampus, parahippocampal gyrus, inferior temporal gyrus, middle temporal gyrus, lingual gyrus, calcarine, inferior occipital gyrus and middle occipital gyrus (Fig. 3). Here, we investigated which of the nine white matter tracts contribute to general semantic processing or modality-specific semantic processing.

### General semantic-relevant connections of the semantic hub

The correlations between the semantic PCA scores and the integrity values of each of the nine connections of the left FFG for each diffusion metric (fractional anisotropy and mean, axial and radial diffusivity) are shown in Table 4. We observed that the integrity values of four connections were significantly associated with the semantic PCA scores for at least two metrics (Fig. 3): the left FFG–left superior temporal pole (*r* < −0.32, FDR-corrected *q*s < 0.01); the left FFG–left middle temporal gyrus (*r* < −0.32, FDR-corrected *q*s < 0.01); the left FFG–left inferior temporal gyrus (*r* < −0.30, FDR-corrected *q*s < 0.01); and the left FFG–left hippocampus (*r* < −0.40, FDR-corrected *q*s < 0.01).

**Table 4:** Correlations between four diffusivity metrics of the tracts and the semantic PCA scores in 79 stroke patients

Diffusion metrics White matter connections	Fractional anisotropy		Mean diffusivity		Axial diffusivity		Radial diffusivity	
	*r*-value	*P-*value	*r*-value	*P*-value	*r*-value	*P*-value	*r*-value	*P*-value
Left FFG–left superior temporal pole	0.21	0.07	−0.33	0.004^a^	−0.32	0.004	−0.32	0.005^a^
Left FFG–left hippocampus	0.29	0.01	−0.41	0.0002^a^	−0.40	0.0003^a^	−0.41	0.0002^a^
Left FFG–left parahippocampal gyrus	−0.11	0.33	−0.27	0.02	−0.27	0.02	−0.26	0.03
Left FFG–left inferior temporal gyrus	0.11	0.36	−0.32	0.005^a^	−0.30	0.007	−0.32	0.005^a^
Left FFG–left middle temporal gyrus	0.18	0.11	−0.34	0.002^a^	−0.34	0.002^a^	−0.32	0.004^a^
Left FFG–left lingual gyrus	0.03	0.81	−0.09	0.45	−0.08	0.48	−0.09	0.45
Left FFG–left calcarine	0.02	0.89	−0.11	0.35	−0.12	0.32	−0.09	0.41
Left FFG–left inferior occipital gyrus	−0.18	0.13	−0.05	0.66	−0.07	0.57	−0.03	0.77
Left FFG–left middle occipital gyrus	0.02	0.87	−0.13	0.25	−0.14	0.23	−0.12	0.30

FFG, fusiform gyrus.

^a^FDR-corrected *q* < 0.01.

After controlling for the confounders, all the tracts still showed a significant correlation with the semantic PCA scores (*P* < 0.05; Supplementary Table 3). This indicated that the significant effects of the observed semantic tracts could not be explained by total grey matter volume or grey matter volume of the node regions and that the effects of these tracts were specific to semantic processing.

### Modality-specific semantic connections of the semantic hub

To explore the modality-specific semantic connections, we correlated the integrity value of each tract of the left FFG with each of the six modality-specific semantic scores. The analyses revealed 22 significant correlations of the tracts (FDR-corrected *q*s < 0.01, significant for at least two metrics; Table 5). In particular, the integrity values of the tracts between the left FFG and the left hippocampus, left superior temporal pole and left middle temporal gyrus correlated significantly with all six modality-specific semantic scores (fractional anisotropy: *r* = 0.29–0.46, FDR-corrected *q*s < 0.01; diffusivity metrics: *r* = −0.52 to −0.29, FDR-corrected *q*s < 0.01). The integrity of the left FFG–left inferior temporal gyrus tract correlated significantly with the colour and manipulation modality-specific scores (fractional anisotropy: *r* = 0.31, FDR-corrected *q* < 0.01; diffusivity metrics: *r* = −0.42 to −0.30, FDR-corrected *q*s < 0.01). Additionally, significant correlations were observed between the integrity of the left FFG–left lingual gyrus tract and motion processing (fractional anisotropy: *r*= 0.37, FDR-corrected *q* < 0.01; diffusivity metrics: *r* = −0.32 to −0.29, FDR-corrected *q*s < 0.01) and the left FFG–left parahippocampal gyrus tracts and manipulation processing (*r* = −0.33 to −0.29, FDR-corrected *q*s < 0.01).

**Table 5:** Correlations between four diffusivity metrics of the tracts and the modality-specific task scores in 79 stroke patients

White matter connections	Correlation coefficients of each modality-specific task					
	Form	Colour	Motion	Sound	Manipulation	Function
Fractional anisotropy						
Left FFG–left superior temporal pole	0.40^a^	0.32^a^	0.36^a^	0.31^a^	0.34^a^	0.29^a^
Left FFG–left hippocampus	0.46^a^	0.40^a^	0.42^a^	0.36^a^	0.44^a^	0.37^a^
Left FFG–left parahippocampal gyrus	0.12	0.02	0.13	0.10	0.16	0.12
Left FFG–left inferior temporal gyrus	0.37^a^	0.26	0.32^a^	0.29^a^	0.31^a^	0.26
Left FFG–left middle temporal gyrus	0.40^a^	0.33^a^	0.37^a^	0.32^a^	0.36^a^	0.30^a^
Left FFG–left lingual gyrus	0.36^a^	0.24	0.37^a^	0.25	0.22	0.21
Left FFG–left calcarine	0.30^a^	0.21	0.34^a^	0.22	0.19	0.15
Left FFG–left inferior occipital gyrus	0.01	−0.04	0.14	−0.07	−0.04	−0.06
Left FFG–left middle occipital gyrus	0.31^a^	0.21	0.35^a^	0.24	0.25	0.19
Mean diffusivity						
Left FFG–left superior temporal pole	−0.29^a^	−0.36^a^	−0.32^a^	−0.33^a^	−0.41^a^	−0.33^a^
Left FFG–left hippocampus	−0.40^a^	−0.45^a^	−0.38^a^	−0.38^a^	−0.50^a^	−0.41^a^
Left FFG–left parahippocampal gyrus	−0.10	−0.20	−0.13	−0.24	−0.32^a^	−0.24
Left FFG–left inferior temporal gyrus	−0.12	−0.30^a^	−0.14	−0.18	−0.40^a^	−0.25
Left FFG–left middle temporal gyrus	−0.28	−0.37^a^	−0.28	−0.36^a^	−0.42^a^	−0.30^a^
Left FFG–left lingual gyrus	−0.21	−0.20	−0.29^a^	−0.28	−0.22	−0.26
Left FFG–left calcarine	−0.14	−0.13	−0.16	−0.19	−0.18	−0.08
Left FFG–left inferior occipital gyrus	0.08	0.05	−0.10	−0.04	−0.08	−0.06
Left FFG–left middle occipital gyrus	−0.09	−0.12	−0.13	−0.18	−0.24	−0.11
Axial diffusivity						
Left FFG–left superior temporal pole	−0.19	−0.30^a^	−0.24	−0.29	−0.33^a^	−0.26
Left FFG–left hippocampus	−0.35^a^	−0.42^a^	−0.33^a^	−0.35^a^	−0.47^a^	−0.37^a^
Left FFG–left parahippocampal gyrus	−0.07	−0.18	−0.11	−0.22	−0.29^a^	−0.20
Left FFG–left inferior temporal gyrus	−0.04	−0.24	−0.07	−0.15	−0.33^a^	−0.18
Left FFG–left middle temporal gyrus	−0.17	−0.30^a^	−0.18	−0.31^a^	−0.33^a^	−0.21
Left FFG–left lingual gyrus	−0.13	−0.15	−0.22	−0.24	−0.17	−0.21
Left FFG–left calcarine	−0.01	−0.04	−0.02	−0.12	−0.11	0.00
Left FFG–left inferior occipital gyrus	0.10	0.09	−0.05	−0.05	−0.08	−0.05
Left FFG–left middle occipital gyrus	0.06	−0.02	0.03	−0.09	−0.13	−0.01
Radial diffusivity						
Left FFG–left superior temporal pole	−0.33^a^	−0.38^a^	−0.34^a^	−0.34^a^	−0.42^a^	−0.36^a^
Left FFG–left hippocampus	−0.43^a^	−0.46^a^	−0.40^a^	−0.39^a^	−0.52^a^	−0.43^a^
Left FFG–left parahippocampal gyrus	−0.12	−0.21	−0.15	−0.24	−0.33^a^	−0.25
Left FFG–left inferior temporal gyrus	−0.16	−0.33^a^	−0.18	−0.20	−0.42^a^	−0.28
Left FFG–left middle temporal gyrus	−0.32^a^	−0.38^a^	−0.31^a^	−0.36^a^	−0.44^a^	−0.33^a^
Left FFG–left lingual gyrus	−0.24	−0.22	−0.32^a^	−0.29	−0.23	−0.28
Left FFG–left calcarine	−0.20	−0.17	−0.23	−0.22	−0.21	−0.13
Left FFG–left inferior occipital gyrus	0.06	0.03	−0.12	−0.03	−0.07	−0.06
Left FFG–left middle occipital gyrus	−0.16	−0.17	−0.21	−0.21	−0.27	−0.16

FFG, fusiform gyrus.

^a^FDR-corrected *q* < 0.01.

Even after controlling for the confounding covariates, most of the correlations remained significant (*P* < 0.05), except for the effects of the left FFG–lingual gyrus, FFG–superior temporal pole and FFG–middle temporal gyrus on motion knowledge and the left FFG–superior temporal pole and FFG–middle temporal gyrus on form knowledge.

## Discussion

Using DWI data and behavioural data from the 79 stroke patients, we tested whether the left FFG was still a semantic hub in stroke patients and investigated the semantic-relevant connections of this hub in the brain network. We confirmed that the left FFG was the semantic hub also in stroke patients, because its nodal degree values in the structural brain network predicted the general semantic processing ability of the patients. The hub region functions together with four other regions (the left superior temporal pole, the middle temporal gyrus, the inferior temporal gyrus and the hippocampus) for general semantic processing. Moreover, we found that the connections between the left FFG and the left hippocampus, left superior temporal pole, left middle temporal gyrus, left inferior temporal gyrus and left parahippocampal gyrus were involved in the processing of object form, colour, motion, sound, manipulation and function knowledge. In sum, the current study verified the results of our previous study on semantic dementia^18^ and provided new evidence for the hub-and-spoke theory of the semantic system in stroke patients.

### Semantic hub: the left FFG

Our results showed that the disconnection of the left FFG from other brain regions may be a factor related to the general semantic deficits of stroke patients. The critical role of the ATL in semantic processing has been demonstrated in several stroke studies supporting the hub-and-spoke model.^10,12,14^ Our research further showed that the left FFG within the ATL was the semantic hub. This is consistent with the findings in previous literature that also pointed to the critical function of the left FFG in semantic processing. For example, functional MRI studies in healthy participants have consistently observed activation of this region in semantic conditions.^24,40,41^ Studies of semantic dementia have also revealed that hypometabolism and atrophy of this region are associated with semantic deficits in patients.^13,42,43^ Furthermore, lesion–symptom mapping studies in stroke patients have evidenced that the left FFG was crucial for semantic fluency.^44,45^ Alyahya *et al.*^46^ reported the association between the left FFG lesion and the semantic processing component extracted from connected speech production in stroke aphasia. Another study found that posterior cerebral artery strokes affecting the left FFG were often associated with semantic damage and showed disproportionate impairment in specific semantic categories.^47^

Our previous study in patients with semantic dementia found that the left FFG is a semantic hub whose disconnection from other regions correlates with general semantic impairment in the cohort.^18^ However, due to the atrophy of the left FFG in these patients, it is difficult to determine whether the semantic deficits are caused by local damage to the left FFG or by disconnection of this region from other regions. The multimodal ‘hub’ region of the ventral ATL, which is the focus of atrophy in semantic dementia, is anatomically shielded from stroke as it receives a dual blood supply.^27,28^ This study shows that although the left FFG of stroke patients is undamaged and functionally intact, the disruption of its connectivity with other brain regions is still related to semantic impairment. Therefore, the left FFG, as a hub region, not only performs semantic processing through its own functions^13,44,46,47^ but also works with other brain regions to influence semantic performance.

Note that the right FFG showed a negative association with the semantic scores (i.e. weaker connectivity strength is associated with better performance). This is inconsistent with the hub-and-spoke theory, which suggests that the semantic knowledge is represented in bilateral ATLs.^6^ The unexpected effect of the right FFG is probably due to the difference between left and right hemisphere stroke patients. Because the left hemisphere stroke patients performed worse and had less damage on the right FFG than the right hemisphere stroke patients, the beta value under fractional anisotropy was negative for the right FFG, and the beta values under three diffusivity metrics were positive for the right FFG. There are two possible explanations for the poorer performance of left hemisphere stroke patients. First, it could be due to the insensitivity of the non-verbal semantic measures. Previous studies have indicated the lateralization of non-verbal semantics in the right hemisphere.^9,48^ However, our patients were less impaired on the non-verbal tasks compared to the verbal tasks (see the *t*-scores in Table 1). Because of the insensitive non-verbal measures, the neural correlate of the non-verbal component, i.e. the right ATL, may be difficult to be detected. Second, the unexpected result might be due to the fact that our behavioural tasks require more processing of concrete object concepts, whereas the right ATL is mainly involved in abstract semantic processing.^9^

### General semantic connectivity of the hub region

In the stroke patients, the connections between the left FFG hub region and the left hippocampus, superior temporal pole, middle temporal gyrus and inferior temporal gyrus were found to be related to the general semantic scores. We have identified important semantic connections from the semantic hub to the temporal lobe (superior temporal pole, inferior temporal gyrus and middle temporal gyrus) as well as the limbic system (hippocampus) in patients with semantic dementia,^18^ which were again validated in the present experiment with stroke patients. This finding provides further support for the hub-and-spoke theory of semantic representation. We speculate that there are functional divisions between the connections. The connection between the left FFG and the temporal pole may be responsible for processing concepts of unique entities.^49-52^ The hippocampus is an important centre for memory processing.^53,54^ The left FFG–hippocampus pathway may activate during concept retrieval, mimicking the process during concept learning, and is responsible for further activation of modality-specific brain regions.^55-57^ The connections between the left FFG and other temporal brain regions may be responsible for the representation of semantic information such as form, colour and sound.^58^

### Modality-specific semantic connectivity of the hub region

Some additional modality-specific semantic connections were found in this study with stroke patients. First, we found that the integrity of the left FFG–hippocampus tract was related to all six modality-specific knowledge processing. The integrity of the left FFG–parahippocampal gyrus tract was also associated with manipulation knowledge processing. The hippocampus and the parahippocampal gyrus have been identified as key brain regions linking episodic and semantic memory.^54,59^ They can function to activate corresponding memory fragments during semantic retrieval.^55-57^ Therefore, it is likely that the left FFG–hippocampus and FFG–parahippocampal gyrus tracts function during the retrieval of semantic information in these modalities because these modalities require the reproduction of episodic memory. Second, we observed the role of the left FFG–superior temporal pole tract in the processing of colour, manipulation, sound and function knowledge. The temporal pole is generally considered to be the brain region that represents the concepts of unique entities, such as specific persons and objects.^49-52^ These four types of modality-specific knowledge may rely on this white matter tract because subjects need to consider the relevant features of the unique entities when invoking relevant modality knowledge. Third, we found that the left FFG–middle temporal gyrus tract was involved in the processing of colour, manipulation, sound and function knowledge. We also observed an association between the left FFG–inferior temporal gyrus tract and the processing of colour and manipulation knowledge. The results are consistent with the existing literature. Previous studies have also shown that the middle temporal gyrus and the inferior temporal gyrus play different roles in the processing of modality knowledge, such as colour,^60^ manipulation^61^ and sound.^62,63^

### Limitations

The study has some limitations. First, although we tried to recruit a more representative sample, it was gender imbalanced, with 82% being male. Also, a considerable number of patients were in the 1–6-month post-stroke period, and patients in the subacute phase may still be recovering. These biases could affect the generalizability and reliability of the results. Second, the distribution of brain damage in our sample was dispersed, and the nodes and tracts with smaller numbers of lesioned patients might be underpowered. Such low power could reduce the chance of detecting true effects and lead to low reproducibility of the results.^64^ Future studies need to validate the current findings in samples with more focused lesions to increase statistical power. Third, the pattern of brain damage is a direct factor affecting the detectability of effects. Some brain areas, such as those in the occipital lobes, remain intact in stroke patients. This could leave potentially important semantic brain regions and white matter connections undetected.

## Conclusion

By examining the relationship between general and modality-specific semantic performance in stroke patients and the nodal degree of the region and the diffusion metrics of the connections, we identified that the left FFG was the semantic hub region supporting general semantic processing through white matter connections with four other brain regions. We also observed that the connections between the semantic hub and the left hippocampus, left superior temporal pole, left middle temporal gyrus, left inferior temporal gyrus and left parahippocampal gyrus were differentially involved in processing form, colour, motion, manipulation, sound and function knowledge. The results of the current study confirmed the role of the left FFG as a semantic hub in stroke patients. The observed semantic connections with the semantic hub provide new evidence for the neuroanatomical organization of semantic memory in the human brain.

## Supplementary Material

fcae058_Supplementary_Data

## Data Availability

The data that support the findings of this study are available from the corresponding author, upon reasonable request.
