# Supplementary material for: White matter network underlying semantic processing: evidence from stroke patients
Source: Brain Commun. 2024 Feb 21;6(2):fcae058. doi: 10.1093/braincomms/fcae058 (PMC10914445; doi:10.1093/braincomms/fcae058)
Supplement: fcae058_Supplementary_Data [file fcae058_supplementary_data.docx]

Supplementary Material

# Supplementary Methods

## Behavioural data collection

We showed an example of additional instructions that were provided to patients and introduced details of modality-specific semantic tasks and non-semantic control tasks.

### Example of additional instructions to patients

We used two methods to ensure that the participants understood the tasks. First, we conducted practice trials with each participant to ensure that they were able to perform the tasks. Second, for each item in the tasks, if a patient showed signs of not understanding the instructions, the experimenter would provide additional guidance. Here is an example from the oral sound naming task. After the sound of hammer was played, the experimenter asked, “What object made the sound?” The patient replied, “Striking.” In this case, the experimenter repeated the instruction and provided guidance by asking “Can you tell me the name of the object that made this sound? What do we use to strike?” The patient then responded “hammer”, the correct answer. In this case, the item was still scored as the original answer could have been due to word retrieval difficulties or a lack of understanding of the instructions.

### Modality-specific semantic tasks

Modality-specific semantic tasks were conducted to assess the subjects’ processing ability on six specific sensorimotor modalities of objects (form, colour, motion, sound, manipulation and function). Each modality included a verbal task and a nonverbal task. In each verbal task, subjects were instructed to choose which of the two object names shown at the bottom was a better answer to the top written question about a given sensorimotor attribute. Verbal modality-specific tasks included: form matching (75 items, e.g. which has four legs? cat or goldfish), colour matching (30 items, e.g. which has the same colour on the whole body? panda or camel), motion matching (30 items, e.g. which can jump? rabbit or tortoise), sound matching (45 items, e.g. which produces a louder sound? dog or mouse), manipulation matching (30 items, e.g. which is primarily used by arms? saw or key), and function attribute matching (60 items, e.g. which is used for transportation? train or castle). For the nonverbal modality-specific tasks, the stimuli were presented as pictures or sounds and the tasks involved no verbal input or output. The nonverbal tasks were as follows: form verification (60 items, two pictures of object parts, e.g. the head of a lion and the body of a zebra, were presented; and subjects were instructed to identify whether the two parts came from the same object), colour verification (20 items, a colour patch and a grayscale object picture appeared on the screen; and subjects needed to determine whether the colour was common for the object), motion verification (57 items, subjects saw a point-light motion animation, e.g. a tortoise climbing, and a pictured object, e.g. a rabbit; and they needed to verify whether the motion was typical for the object), sound verification (42 items, an object picture, e.g. a sheep, and a sound of an object, e.g. cat’s cry, were presented; and subjects had to determine whether the sound was typical for the object in the picture), manipulation matching (20 items, subjects were instructed to judge which of the two objects shown at the bottom was more similar to the object shown at the top in terms of typical manner of manipulation), function matching (30 items, subjects had to determine which of the two objects shown at the bottom had a similar function with the object shown at the top of the screen).

### Non-semantic control tasks

To control for the influence of non-semantic processing ability such as primary perception and executive control, we assessed the following tasks that involved no or minimal semantic processing: visual perception (30 items, subjects were instructed to determine whether two black circles had the same size), sound perception (44 items, subjects needed to determine whether two episodes of sound had the same rhythm and pitch), and number proximity matching (3 items, subjects were instructed to determine which of the two numbers was quantitatively closer to a third number shown at the top).

## Imaging data collection

3D T1 images were obtained with a magnetisation prepared rapid gradient echo (MPRAGE) sequence along the sagittal plane with the following parameters: repetition time = 12.26 ms, echo time = 4.2 ms, flip angle = 15°, matrix size = 512 × 512, field of view = 250 mm × 250 mm, slice number = 248 slices, slice thickness = 0.70 mm, voxel size = 0.49 mm × 0.49 mm × 0.70 mm. FLAIR T2-weighted images were scanned along the axial plane with parameters: repetition time = 8003 ms, echo time = 127.57 ms, flip angle = 90°, matrix size = 512 × 512, field of view = 250 mm ×250 mm, slice number = 28 slices, slice thickness = 5 mm, voxel size = 0.49 mm × 0.49 mm × 5 mm.

# Supplementary Results

## Brain damage of stroke patients

Given that the purpose of this study was to investigate whether the semantic hub region identified in semantic dementia (i.e. the left FFG)^1^ could also be identified in patients with no injury in that region (i.e. the stroke patients), it was necessary to compare the brain injury patterns and left FFG damage between patients with semantic dementia and stroke patients. We present in Supplementary Table 2 the percentage of grey matter damage in each AAL brain region in 33 semantic dementia patients and in 79 stroke patients, respectively. The semantic dementia cohort was identical to that in the study of Chen and colleagues.^1^ The results indicated that patients with semantic dementia had severe left FFG damage (20%), while the stroke patients showed no damage in this region (0%). Thus, we could rule out damage to the left FFG itself in stroke patients and explore if its connectivity (nodal degree values) is still predictive of the patient’s semantic performance.

**Verifying the semantic hub region in patients with sematic deficit**

We conducted the same regression analyses in two subgroups of semantic deficit patients, characterised as those with general semantic scores below the mean score and below the mean score minus 0.5 standard deviation of the healthy control group. For each patient subgroup, the results of the regression analyses were consistent with that included all 79 patients [fractional anisotropy: right fusiform gyrus (beta values = -0.51 to -0.50), left triangular part of inferior frontal gyrus (beta values = 0.35 to 0.43); mean diffusivity: right fusiform gyrus (beta values = 0.42 to 0.47), left fusiform gyrus (beta value = -0.28); axial diffusivity: right fusiform gyrus (beta values = 0.40 to 0.45), left fusiform gyrus (beta values = -0.29 to -0.25); radial diffusivity: right fusiform gyrus (beta values = 0.43 to 0.45), left fusiform gyrus (beta value = -0.28)]. These validation analyses further confirmed the role of the left FFG as the semantic hub.

# Supplementary Discussion

We used PCA to assess the semantic processing ability of stroke patients and identified three components. Note that the perceptual component had relatively high loading weights on three of the general semantic tasks (i.e. picture associative matching, word associative matching, and word-picture verification; Table 2). We suspect that this may be due to the fact that these tasks involve a larger number of items and require a more careful comparison between the items than the other three general semantic tasks. Considering the higher processing demands resulting from a larger number of inputs and the more careful comparison between them, these tasks might require more perceptual processing.


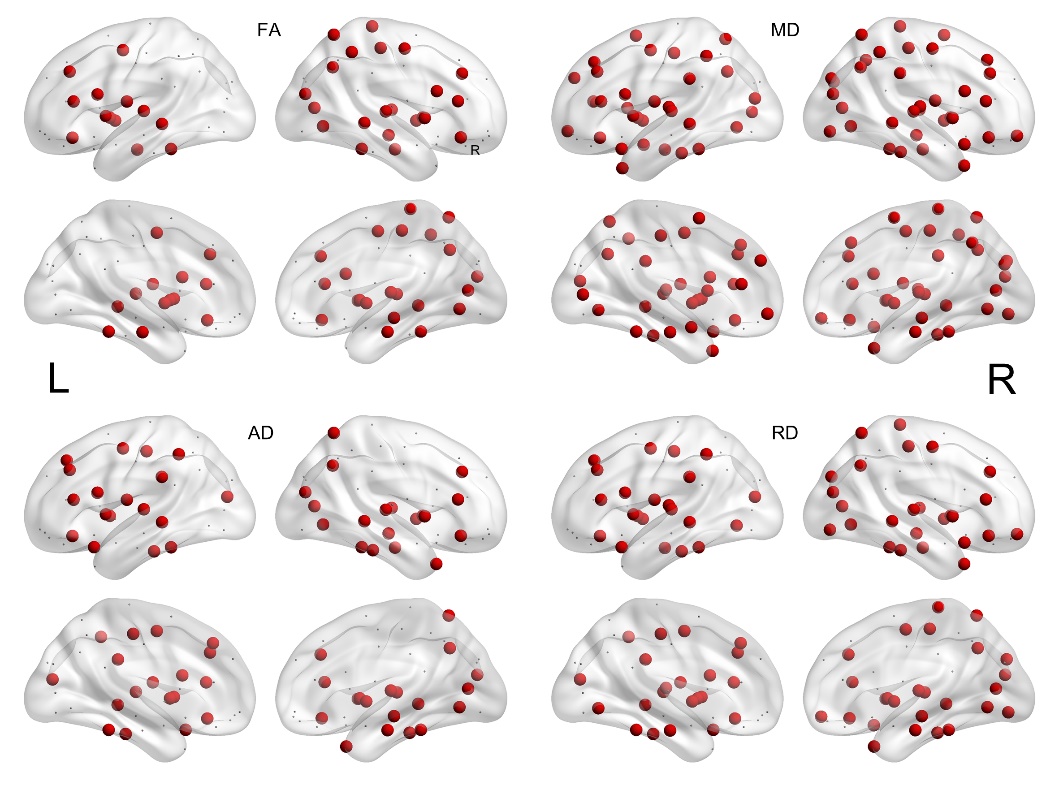


**Supplementary Figure 1. Correlation between nodal degree values of AAL regions and the semantic performance in stroke patients.** Red dots represented the regions whose nodal degree values significantly correlated with the semantic PCA scores under each metric (FDR corrected *qs* < 0.01). AD = axial diffusivity; FA = fractional anisotropy; MD = mean diffusivity; RD = radial diffusivity.

**Supplementary Table 1 Background information of 79 stroke patients.**

| **Code** | **Gender** | **Age**  **(year)** | **Education**  **(year)** | **Post-onset duration**  **(month)** | **Etiology** | **Lesion site** | **Type of aphasia** |
| --- | --- | --- | --- | --- | --- | --- | --- |
| 4 | male | 49 | 16 | 6 | haemorrhage | left hemisphere | motor |
| 7 | male | 48 | 15 | 4 | infarction | left hemisphere | global |
| 8 | male | 60 | 16 | 6 | haemorrhage | left hemisphere | global |
| 9 | male | 43 | 19 | 11 | infarction | right hemisphere | NO |
| 16 | male | 32 | 15 | 9 | haemorrhage | left hemisphere | global |
| 18 | male | 34 | 12 | 10 | haemorrhage | left hemisphere | anomia |
| 19 | male | 42 | 14 | 6 | infarction | left hemisphere | global |
| 21 | male | 63 | 12 | 1 | infarction | right hemisphere | anomia |
| 22 | male | 49 | 12 | 3 | infarction | left hemisphere | global |
| 23 | male | 60 | 16 | 2 | infarction | left hemisphere | NO |
| 25 | male | 46 | 16 | 2 | infarction | right hemisphere | motor |
| 27 | male | 58 | 12 | 6 | infarction | right hemisphere | sensory |
| 29 | male | 30 | 19 | 3 | infarction | right hemisphere | conduction |
| 30 | male | 41 | 15 | 8 | haemorrhage | left hemisphere | anomia |
| 33 | male | 36 | 15 | 10 | haemorrhage | left hemisphere | anomia |
| 34 | female | 64 | 12 | 5 | infarction | left hemisphere | NO |
| 35 | male | 46 | 12 | 6 | infarction | right hemisphere | subcortical |
| 38 | male | 40 | 12 | 3 | haemorrhage | right hemisphere | sensory |
| 42 | male | 45 | 12 | 3 | haemorrhage | right hemisphere | conduction |
| 48 | female | 56 | 12 | 6 | haemorrhage | right hemisphere | motor |
| 56 | male | 61 | 15 | 15 | haemorrhage | right hemisphere | NO |
| 57 | male | 51 | 9 | 2 | infarction | left hemisphere | motor |
| 60 | male | 45 | 16 | 4 | infarction | left hemisphere | anomia |
| 62 | female | 56 | 12 | 6 | infarction | left hemisphere | global |
| 66 | male | 35 | 16 | 2 | infarction | right hemisphere | NO |
| 67 | male | 47 | 9 | 5 | infarction | right hemisphere | anomia |
| 68 | male | 40 | 16 | 6 | haemorrhage | left hemisphere | subcortical |
| 79 | male | 48 | 19 | 24 | haemorrhage | right hemisphere | NO |
| 82 | female | 35 | 15 | 16 | haemorrhage | left hemisphere | conduction |
| 83 | male | 54 | 16 | 4 | infarction | right hemisphere | motor |
| 85 | male | 67 | 9 | 7 | infarction | left hemisphere | motor |
| 89 | male | 46 | 9 | 2 | infarction | left hemisphere | global |
| 91 | male | 65 | 9 | 8 | infarction | right hemisphere | sensory |
| 97 | male | 55 | 15 | 3 | infarction | right hemisphere | motor |
| 98 | male | 62 | 12 | 10 | infarction | right hemisphere | subcortical |
| 101 | male | 35 | 16 | 2 | infarction | left hemisphere | global |
| 102 | male | 28 | 16 | 3 | infarction | left hemisphere | global |
| 103 | female | 37 | 12 | 3 | infarction | left hemisphere | sensory |
| 104 | male | 68 | 16 | 2 | infarction | left hemisphere | global |
| 106 | female | 22 | 16 | 2 | haemorrhage | right hemisphere | NO |
| 109 | male | 58 | 9 | 7 | infarction | left hemisphere | global |
| 111 | female | 51 | 8 | 5 | infarction | right hemisphere | motor |
| 115 | male | 20 | 9 | 2 | haemorrhage | left hemisphere | anomia |
| 116 | male | 56 | 15 | 5 | infarction | left hemisphere | motor |
| 117 | female | 45 | 15 | 2 | infarction | right hemisphere | sensory |
| 118 | female | 40 | 12 | 6 | infarction | left hemisphere | global |
| 122 | male | 63 | 12 | 2 | infarction | left hemisphere | global |
| 124 | male | 52 | 12 | 3 | infarction | right hemisphere | anomia |
| 125 | male | 48 | 9 | 3 | haemorrhage | right hemisphere | sensory |
| 126 | male | 48 | 9 | 2 | infarction | left hemisphere | motor |
| 129 | male | 58 | 15 | 1 | infarction | left hemisphere | global |
| 132 | male | 37 | 12 | 3 | infarction | left hemisphere | global |
| 133 | male | 47 | 15 | 3 | infarction | right hemisphere | sensory |
| 159 | female | 39 | 15 | 3 | haemorrhage | right hemisphere | sensory |
| 161 | male | 53 | 12 | 1 | infarction | left hemisphere | motor |
| 162 | male | 48 | 15 | 3 | haemorrhage | left hemisphere | conduction |
| 163 | male | 47 | 16 | 2 | infarction | left hemisphere | global |
| 165 | male | 47 | 16 | 5 | haemorrhage | left hemisphere | global |
| 169 | male | 39 | 12 | - | haemorrhage | right hemisphere | motor |
| 170 | male | 43 | 16 | 16 | infarction | left hemisphere | motor |
| 177 | male | 26 | 16 | 3 | infarction | left hemisphere | global |
| 180 | female | 41 | 15 | 5 | haemorrhage | right hemisphere | sensory |
| 181 | male | 49 | 15 | 5 | haemorrhage | left hemisphere | anomia |
| 182 | female | 40 | 15 | 3 | infarction | right hemisphere | NO |
| 183 | male | 36 | 16 | - | haemorrhage | left hemisphere | global |
| 185 | male | 57 | 6 | 2 | infarction | left hemisphere | subcortical |
| 190 | male | 42 | 12 | 6 | haemorrhage | left hemisphere | anomia |
| 191 | male | 28 | 16 | 3 | haemorrhage | left hemisphere | global |
| 194 | male | 40 | 16 | 2 | haemorrhage | right hemisphere | conduction |
| 206 | male | 40 | 15 | 2 | haemorrhage | left hemisphere | global |
| 209 | male | 48 | 9 | 2 | haemorrhage | left hemisphere | global |
| 210 | male | 74 | 12 | 3 | haemorrhage | right hemisphere | sensory |
| 211 | female | 52 | 6 | 3 | infarction | right hemisphere | motor |
| 213 | male | 55 | 15 | 2 | haemorrhage | left hemisphere | global |
| 217 | female | 34 | 15 | 2 | infarction | left hemisphere | motor |
| 226 | male | 45 | 15 | 5 | infarction | left hemisphere | global |
| 231 | male | 43 | 15 | 4 | infarction | right hemisphere | dysgraphia |
| 304 | male | 70 | 12 | 2 | infarction | left hemisphere | global |
| 305 | male | 47 | 12 | 4 | infarction | left hemisphere | global |

The type of aphasia was assessed using Neuropsychological test of Chinese aphasia^2^. “NO” indicated that the patient did not exhibit symptoms of aphasia.

**Supplementary Table 2 Mean percentage of damage in each AAL brain region in patients with semantic dementia versus stroke.**

| **Percentage of damage in each region in semantic dementia** | | | | | |  | **Percentage of damage in each region in stroke patients** | | | | | |
| --- | --- | --- | --- | --- | --- | --- | --- | --- | --- | --- | --- | --- |
| **Regions** | **PERC** | **Regions** | **PERC** | **Regions** | **PERC** |  | **Regions** | **PERC** | **Regions** | **PERC** | **Regions** | **PERC** |
| lTPOmid | 43% | rSTG | 13% | rMFG | 5% |  | lINS | 21% | lCAU | 4% | lSFGdor | 1% |
| rTPOmid | 42% | rORBinf | 12% | lROL | 5% |  | lPUT | 20% | lMFG | 4% | lSPG | 1% |
| lTPOsup | 37% | rORBsup | 12% | rIPL | 5% |  | rPUT | 16% | rPreCG | 4% | rACG | 1% |
| rTPOsup | 35% | rPAL | 12% | rROL | 5% |  | lROL | 16% | rPoCG | 4% | rORBsup | 1% |
| lAMYG | 34% | lPAL | 12% | lSMA | 5% |  | lHES | 14% | rTPOmid | 4% | rPCG | 1% |
| rAMYG | 34% | lHES | 11% | rIFGope | 5% |  | lIFGope | 14% | rANG | 3% | rCUN | 1% |
| lITG | 29% | lSTG | 11% | lSMG | 4% |  | rINS | 13% | lTPOmid | 3% | rSFGdor | 1% |
| rPHG | 27% | rTHA | 11% | rSMA | 4% |  | rHES | 11% | rIPL | 3% | rFFG | 1% |
| lPHG | 27% | lORBsup | 11% | lCUN | 4% |  | rROL | 11% | lORBmid | 3% | rLING | 0% |
| rITG | 27% | lORBsup | 10% | lSOG | 4% |  | lSTG | 11% | lIPL | 3% | lSFGmed | 0% |
| lHIP | 24% | lTHA | 10% | lPreCG | 3% |  | lPAL | 9% | lITG | 3% | lPCG | 0% |
| rHIP | 23% | lORBinf | 9% | lPoCG | 3% |  | lIFGtri | 9% | rMOG | 2% | rPHG | 0% |
| rOLF | 22% | rPCG | 9% | lSPG | 3% |  | rSTG | 9% | rITG | 2% | rREC | 0% |
| lOLF | 22% | rDCG | 9% | rSPG | 3% |  | lORBinf | 8% | rSPG | 2% | lREC | 0% |
| lFFG | 20% | lANG | 8% | rPCUN | 3% |  | lMTG | 8% | lTHA | 2% | lDCG | 0% |
| rMTG | 20% | rANG | 8% | rPoCG | 2% |  | rPAL | 8% | rSOG | 1% | rSFGmed | 0% |
| rFFG | 19% | rORBmid | 8% | rMOG | 2% |  | lSMG | 8% | rMFG | 1% | lCAL | 0% |
| rREC | 19% | lDCG | 8% | lPCUN | 2% |  | rIFGope | 7% | lOLF | 1% | lPHG | 0% |
| lMTG | 19% | lSFGdor | 7% | rSOG | 2% |  | lTPOsup | 7% | rORBmid | 1% | rORBsup | 0% |
| rINS | 18% | lIPL | 7% | lMOG | 2% |  | lAMYG | 7% | rOLF | 1% | lSMA | 0% |
| lREC | 17% | lIFGope | 7% | lPCL | 2% |  | lPreCG | 6% | rHIP | 1% | rIOG | 0% |
| rCAU | 16% | lIFGtri | 7% | rPreCG | 1% |  | rTPOsup | 6% | rDCG | 1% | lPCUN | 0% |
| lINS | 15% | rSMG | 7% | rCUN | 1% |  | rCAU | 6% | rTHA | 1% | lACG | 0% |
| lCAU | 15% | rIFGtri | 7% | lLING | 1% |  | rSMG | 5% | rPCL | 1% | lIOG | 0% |
| lACG | 14% | lSFGmed | 6% | lCAL | 1% |  | lPoCG | 5% | lMOG | 1% | lFFG | 0% |
| rACG | 14% | rSFGmed | 6% | rPCL | 1% |  | rMTG | 5% | rSMA | 1% | lSOG | 0% |
| rORBsup | 14% | rSFGdor | 6% | rIOG | 1% |  | rAMYG | 4% | lORBsup | 1% | lPCL | 0% |
| rPUT | 14% | lMFG | 6% | lIOG | 0% |  | rIFGtri | 4% | rPCUN | 1% | lLING | 0% |
| lPUT | 13% | lORBmid | 6% | rCAL | -1% |  | rORBinf | 4% | lHIP | 1% | lCUN | 0% |
| rHES | 13% | lPCG | 5% | rLING | -2% |  | lANG | 4% | rCAL | 1% | lORBsup | 0% |

The value of semantic dementia was calculated as 100% - patient’s grey matter volume in that region / healthy subject’s grey matter volume in that region. The value of stroke patients was calculated as the number of damaged voxels in that brain region / total voxels in that brain region. lFFG = left fusiform gyrus; PERC = percentage.

**Supplementary Table 3 Partial correlation coefficients between the integrity values of the tracts and the semantic PCA scores in 79 stroke patients.**

| **Diffusion metrics White matter connections** | **Fractional anisotropy** | |  | **Mean diffusivity** | |  | **Axial diffusivity** | |  | **Radial diffusivity** | |
| --- | --- | --- | --- | --- | --- | --- | --- | --- | --- | --- | --- |
|  | ***r*-value** | ***P*-value** |  | ***r*-value** | ***P*-value** |  | ***r*-value** | ***P*-value** |  | ***r*-value** | ***P*-value** |
| Left FFG-left superior temporal pole | 0.15 | 0.22 |  | -0.25 | 0.04* |  | -0.26 | 0.03* |  | -0.23 | 0.05* |
| Left FFG-left hippocampus | 0.18 | 0.13 |  | -0.34 | 0.004** |  | -0.34 | 0.004** |  | -0.33 | 0.004** |
| Left FFG-left inferior temporal gyrus | 0.02 | 0.87 |  | -0.24 | 0.04* |  | -0.24 | 0.05* |  | -0.23 | 0.05* |
| Left FFG-left middle temporal gyrus | 0.11 | 0.37 |  | -0.22 | 0.07 |  | -0.24 | 0.05* |  | -0.20 | 0.10 |

Partial correlations between four diffusivity metrics of the general semantic correlation tracts and the semantic PCA scores in the stroke patients after additionally controlling for potential confounding factors (whole-brain lesion volume, lesion volume of the left fusiform gyrus, lesion volume of the other node, and scores on three non-semantic control tasks). FFG = fusiform gyrus. ***P* < 0.01, **P* < 0.05.

# Reference

1. Chen Y, Huang L, Chen K, *et al*. White matter basis for the hub-and-spoke semantic representation: Evidence from semantic dementia. *Brain*. 2020;143(4):1206-1219.
2. Gao S, Wang Y, Shi S, Liu J, Lin G, Rao B. Standard Aphasia Battery of Chinese. *Chinese Mental Health Journal*. 1992;6(3):125-128.
